# Ultrasound image measurements of erector spinae muscle thickness at four spinal levels in adolescents with idiopathic scoliosis: reliability and concave-convex comparison

**DOI:** 10.1186/1748-7161-8-S2-O36

**Published:** 2013-09-18

**Authors:** Alan Richter, Eric C Parent, Gregory Kawchuk, Marc Moreau, Douglas Hedden, Edmond Lou

**Affiliations:** 1 University of Alberta, Alberta, Canada

## Background

Muscular characteristics in scoliosis are insufficiently documented. Ultrasound imaging measurements of extensor muscle thickness are commonly used in low back pain (LBP)[1] but not in scoliosis. Taking measurements of extensor muscle thickness may aid in exercise prescription for patients with adolescent idiopathic scoliosis (AIS)[2].

## Purpose

The purpose of this study is to (1) determine the intra-rater reliability of erector spinae ultrasound thickness measurements at different spinal levels, and (2) determine the concave-convex differences in erector spinae thickness in patients with AIS. 

## Methods

Nine patients with AIS (8 females) with a single thoracic curve, aged 13.5±1.8 years old, with mean Cobb angles of 39.4±9.1o, under observation or undergoing brace treatment were included. In a prone position, three ultrasound images of erector thickness were obtained on each side at L3, the upper end vertebra, lower-end vertebra and the apex of the curve in random order. A 5cm curvilinear probe was used to capture images of the erectors parasagittally over the facets. Thickness was measured using ImageJ as the distance from the facet to the first fascia line by an examiner blinded to image location and measurements. Reliability was estimated using Intraclass Correlation Coefficients (ICC2,1 and 2,2) and standard error of measurement (SEM). Differences between sides were determined using paired t-tests at each level. 

## Results

The intrarater ICCs(2,1) for a single measurement varied between 0.75 and 0.99. The ICCs(2,2) corresponding to the average of the most similar two out of three measurements varied between 0.86 and 0.99, depending on levels. The corresponding SEM for these average measurements varied between 0.03 and 0.17cm (mean 0.09), depending on sides and levels with no systematic pattern. 

The only statistically significant difference between sides was observed at the upper-end vertebra (concave<convex 0.23±0.22cm). Mean extensor thickness was 1.75±0.30cm and 1.98±0.34cm at the left and right upper-end vertebra level, respectively. Mean thickness was 2.33±0.11, 2.18±0.14, and 2.57±0.12cm at the apex, lower-end vertebra and L3, respectively. 

## Conclusions and discussion

Adequate intra-rater reliability for research was obtained by averaging the most similar two of three erector spinae thickness measurements.3 Reliability was similar at all spinal levels and consistent with results in LBP[1]. By measuring at rest, only one small convex-concave thickness difference was detected.
